# Progress in cancer neuroscience

**DOI:** 10.1002/mco2.431

**Published:** 2023-11-22

**Authors:** Yu‐Long Lan, Shuang Zou, Wen Wang, Qi Chen, Yongjian Zhu

**Affiliations:** ^1^ Department of Neurosurgery Second Affiliated Hospital, School of Medicine, Zhejiang University Hangzhou Zhejiang China; ^2^ Key Laboratory of Precise Treatment and Clinical Translational Research of Neurological Diseases Hangzhou Zhejiang China; ^3^ Clinical Research Center for Neurological Diseases of Zhejiang Province Hangzhou China; ^4^ Key Laboratory of Neuropharmacology and Translational Medicine of Zhejiang Province, School of Pharmaceutical Science Zhejiang Chinese Medical University Hangzhou China; ^5^ Department of Neurosurgery Beijing Tiantan Hospital, Capital Medical University Beijing China

**Keywords:** cancer, cancer neuroscience, interaction, neuron, neuroscience, treatment

## Abstract

Cancer of the central nervous system (CNS) can crosstalk systemically and locally in the tumor microenvironment and has become a topic of attention for tumor initiation and advancement. Recently studied neuronal and cancer interaction fundamentally altered the knowledge about glioma and metastases, indicating how cancers invade complex neuronal networks. This review systematically discussed the interactions between neurons and cancers and elucidates new therapeutic avenues. We have overviewed the current understanding of direct or indirect communications of neuronal cells with cancer and the mechanisms associated with cancer invasion. Besides, tumor‐associated neuronal dysfunction and the influence of cancer therapies on the CNS are highlighted. Furthermore, interactions between peripheral nervous system and various cancers have also been discussed separately. Intriguingly and importantly, it cannot be ignored that exosomes could mediate the “wireless communications” between nervous system and cancer. Finally, promising future strategies targeting neuronal–brain tumor interactions were reviewed. A great deal of work remains to be done to elucidate the neuroscience of cancer, and future more research should be directed toward clarifying the precise mechanisms of cancer neuroscience, which hold enormous promise to improve outcomes for a wide range of malignancies.

## Graphical abstract

Neurons and cancer cells’ synaptic communication can modulate cancer growth via neurotransmitters and voltage‐mediated mechanisms. Besides, paracrine signaling between cancer and nerve cells, for instance, the neuron‐mediated secretion of growth factors or neurotransmitters, modulates cancer growth in various tissues. The neuronal influence on malignant cells might be direct or affect other cells in TME. Cancer‐induced paracrine factors modulate nervous system to enhance neural activity in TME. Furthermore, cancers functionally control neural networks, and aberrant neural circuits stimulate tumor progression. Circulating factors released from cancer effects nervous system activity, whereas nervous system influences cancer growth by circulating molecules (hormones and progenitor cells) and alters immune system function.

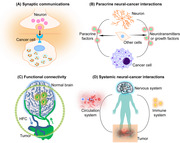

## 1 INTRODUCTION

Even with years of substantial research, the pathophysiological pathways responsible for developing nervous system cancer remain incompletely elucidated. The literature from “cancer neuroscience,” an emerging subject, suggests a close association between nervous system and cancer, which is responsible for developing and growing various brain tumors (BT),^1^, ^2^, ^3^ as well as various other peripheral system tumors. This potential field of “cancer neuroscience” encompasses systemic and local interactions between cancers and the key components of the nervous system—neurons, microglia, oligodendrocytes, astrocytes (ACs), Schwann cells, and peripheral nerves, as well as the various effects of these interactions on the initiation and progression of cancer, the tumor immune microenvironment, and metastasis.^3^

As the nervous system governs such wide‐ranging functions of the human body both in health and disease conditions, it is surprising that it took so long to fully appreciate its essential involvement in cancer. Since the nervous system modulates the homeostasis, development, plasticity, and regeneration in various tissues, scientists are exploring if the nervous system plays similar activity and dictates cancer formation and development.^3^ Various investigations have indicated that nervous system significantly influences tumor invasion and regulates neuronal circuits in cancer.^4^ It is postulated that co‐opted neuronal signaling circuits in tumor cells establish a specific and efficient modulatory mechanism for tumor initiation and malignant advancement. Furthermore, it is accepted that various heterotypic cells populate most tumor environments and form cancer hallmarks, which can expand to acquire neuronal innervation constituting a functionally influential tumor microenvironment (TME).^4^ Therefore, these two specific nervous system interactions substantially contribute to hallmark cancer phenotypes. Cancer cells’ co‐opted neuronal signaling and tumor innervation are believed to regulate cancer advancements and corresponding parameters, such as phenotypic plasticity.^5^ As cancer initiation and progression subvert and repurpose mechanisms of development and regeneration, the nervous system may be implicated in all aspects of cancer pathophysiology. Reciprocally, cancer and relevant therapies could also influence or remodel the nervous system, contributing to pathological feedback loops that could yield neurological dysfunction and also drive malignancy together.^5^ Progress in this intersection between neurobiology and cancer biology may create an important new pillar of cancer therapy.

However, there are a lot of questions and future requirements that need elucidation. And the purposes of this review is providing more clues for answering these questions. Importantly, we need to better map the nervous system–cancer interactome and connectome on multiple levels and scales. This could be essential for gaining deeper insight into the intriguing and complex world of cancer neuroscience. The future neuroscience‐instructed cancer therapies would depend on feasible biomarkers for nervous system–cancer interactions, as well as our ability to conduct meaningful clinical trials. Besides, with more and more mechanisms from various cancers reported, the question arises whether nervous system–cancer interactions may someday be regarded as another general principle of cancer pathogenesis. In the near future, we could expect exciting more in‐depth research in mechanisms known to be relevant for cancer neuroscience today. Furthermore, a better understanding of the role of central nervous system (CNS) and peripheral nervous system (PNS) glial cells and the influence of innervation on various other components of the TME may be required for further complementing our knowledge. Another future requirement will be the joint development of collaborative networks, as well as cross‐disciplinary methodologies, and treatment strategies. To provide more clues for answering these questions, current review aims to systematically discuss the interactions between neurons and cancers and elucidates potential new therapeutic avenues.

In this review, we will exert an update on the current knowledge and future research directions of cancer neuroscience. We consider interactions with the nervous system in both CNS cancers and cancers outside the CNS. The scope of the discussing contents of current review comprises local and systemic interactions between cancer cells and the fundamental components of the nervous system—neurons, non‐neuronal cells, and peripheral nerves, and the effects of these interactions on cancer progression and TME. Besides, we also identify unanswered questions and current roadblocks for potential future clinical translation. The multifactorial connectivity of neurobiology to cancer malignancy described in the current review is provocative and allows future exploration and experimental validation of various human cancers. This review highlights emerging principles, discusses undetermined facts, and underlines the scope of “cancer neuroscience.”

## 2 SUMMARY OF GENERAL INTERACTIONS BETWEEN NERVOUS SYSTEM AND BRAIN CANCERS

**FIGURE 1:**
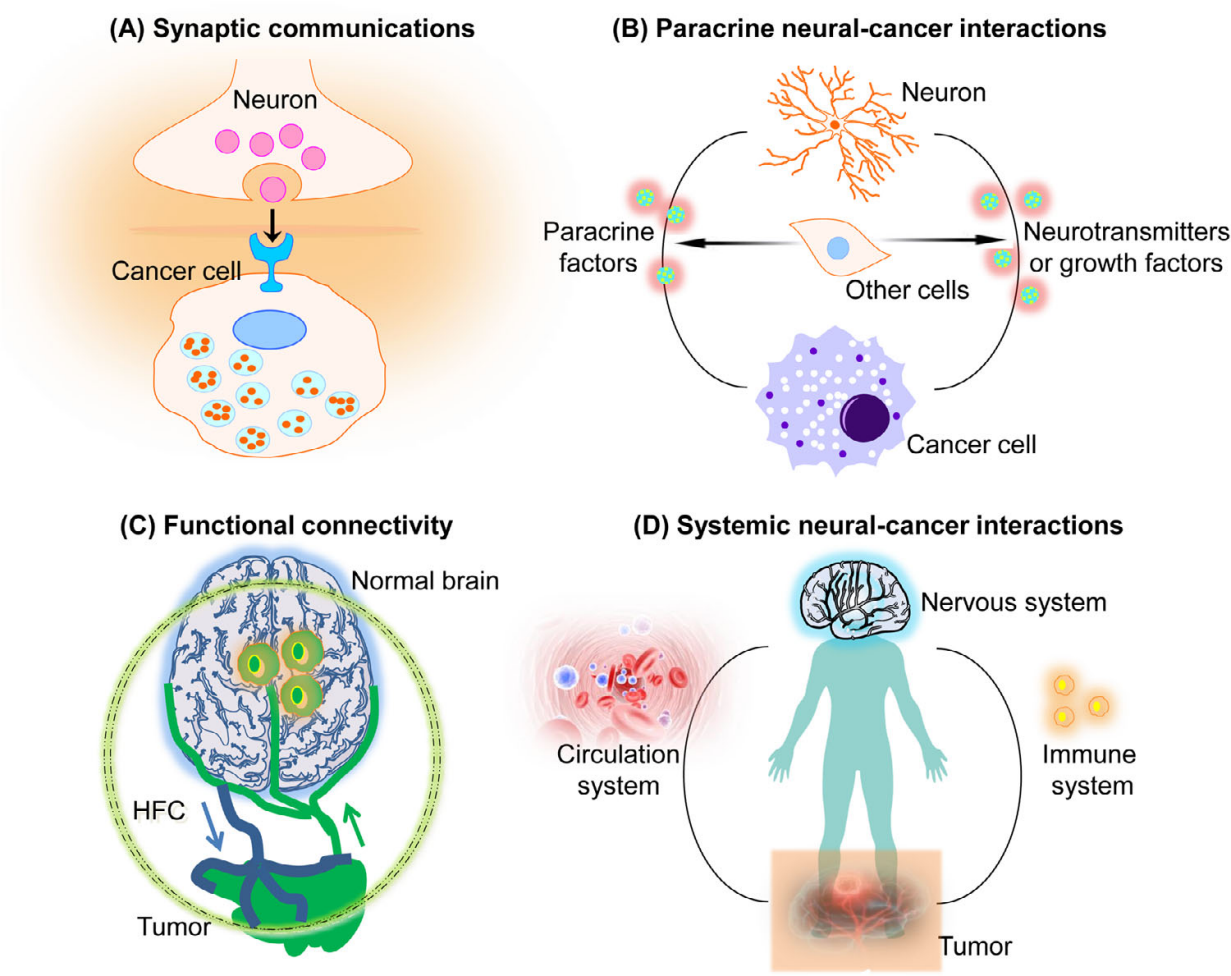
The general concept of nervous system and brain cancer interaction. (A) Neurons and brain cancer cells’ synaptic communication can modulate cancer growth via neurotransmitters and voltage‐mediated mechanisms. Whether synaptic communication between neural axons and cancer cells in peripheral nervous system occurs requires assessment. (B) Paracrine signaling between cancer and nerve cells, for instance, the neuron‐mediated secretion of growth factors or neurotransmitters, modulates cancer growth in various tissues. The neuronal influence on malignant cells might be direct or affect other cells in TME. Cancer‐induced paracrine factors modulate nervous system to enhance neural activity in TME. (C) In the human brain, high‐grade GBMs functionally control neural networks, and aberrant neural circuits stimulate tumor progression. The extent of functional connectivity between normal brain and glioblastoma adversely influences patient survival. Glioblastoma patients exhibit functional connectivity between the tumor and the rest of the brain. Patients without high functional connectivity (HFC) had a shorter overall survival. (D) Circulating factors released from cancer effects nervous system activity, whereas nervous system influences cancer growth by circulating molecules (hormones and progenitor cells) and alters immune system function.

Although the neural cells’ molecular mechanisms that influence cancer cells differ for different tissues, the functional influence of neural cancer communication is predictable by observing the nervous system's influence on its normal cellular counterpart.^3^ This principle can be observed by parallel neuronal influences on normal and neoplastic glial cell growth. In the CNS of the experimental model, glutamatergic neurons stimulate the proliferation of glial precursor cells^6^ and malignant glioblastomas (GBMs).^7^ The underlying process comprises direct electrochemical interaction and paracrine signaling (Figures 1A and B). Neuron‐dependent release of growth molecules and neuronal‐activity‐sensing glial cells stimulates GBM growth.^8^ Furthermore, malignant cells electrically innervate neural networks via neuron‐to‐GBM synapses.^7^ Gap junctions bond with GBM cells, propagating neuronal currents via an interconnected neural‐GBM circuit.^7^, ^9^ Depolarization of GBM cell membrane potential causes postsynaptic electrical signaling to promote cancer development^7^ and subsequent voltage‐sensitive mechanisms, which require further elucidation.

The nervous system crosstalk with cancer through direct interactions and by regulating other cells in TME (such as immune cells, endothelial cells, etc.). This interaction can take place in nerves/neurons in the local microenvironment or in systemic signalings, such as that of elevated neurotransmitter catecholamines (Figure 1C). The nervous system influences the tumor environment through neural angiogenesis regulation via endothelial cell metabolism^10^ or immune system function.^11^ However, interdisciplinary research comprising oncology, immunology, and neuroscience is required for a comprehensive understanding of these essential neural–immune–cancer communications (Figure 1C). Since nervous system–cancer crosstalk is bidirectional, cancers also affect the nervous system's activity, including remodeling and dysfunction. BT (GBMs) signals influence invaded neural network functions by causing abnormal synaptogenesis, enhancing neuronal excitability, and promoting seizures.^12^ Enhanced pathological neuronal activity triggers activity‐dependent signals causing GBM growth.^7^, ^9^ Several tumors have been shown to promote axonogenesis by secreting neurotrophins (e.g., nerve growth molecules), often via a feed‐forward pathway stimulated by enhanced cholinergic or adrenergic signaling.^13^ Furthermore, in TME, neurogenesis from neural precursor cells (NPCs) has also been identified.^14^ Cancers can also invade nerve fibers. In peripheral and central cancers, the functional and structural nervous system remodeling enhances neuron–cancer communication and promotes cancer and related symptoms.

Significantly, the connections between the neurological system and cancer also pertain to tumor forms beyond the CNS, which, unfortunately, have received less attention in research. The cancer‐promoting impact of excitatory neurotransmission also applies to brain metastases. Breast cancer cells that have spread to the brain exhibit an increase in the expression of neurotransmitter receptors and extend perisynaptic processes to receive neurotransmitter signals that are dependent on neuronal activity. These signals activate a signaling cascade mediated by receptors, resulting in the induction of inward currents in the cancerous cells. Consequently, this process promotes the expansion of breast cancer brain metastases.^15^ The mechanisms behind the potential interactions between various forms of metastatic cancer and CNS neurons have yet to be fully elucidated. Furthermore, in experimental model systems, it has been observed that neurotransmitter and growth factor signaling produced from peripheral nerves also have a role in regulating the development of several malignancies, such as gastric, pancreatic, breast, prostate, colon, skin, and oral cancers.^13^, ^16^, ^17^ The communication that occurs among parasympathetic, sympathetic, or sensory neurons inside the TME and malignant cells has the potential to influence the onset, advancement, or spread of cancer, frequently via signaling cascades that rely on neurotransmitters. The understanding of the role of a certain type of nerve is contingent upon the precise context in which it operates. For instance, parasympathetic (also known as cholinergic) nerves can elicit contrasting impacts on various types of tumor tissues. They may stimulate the development of one type of cancer while impeding growth in another type. Regarding this matter, it has been shown that cholinergic signaling has an inhibitory effect on the growth and advancement of pancreatic adenocarcinoma.^17^ However, it has been found to significantly increase adenocarcinoma of the stomach,^13^ which is an organ mostly dominated by parasympathetic innervation. The current understanding regarding the nature of peripheral nerve–cancer cell contacts remains inconclusive since it is uncertain if these interactions solely involve paracrine signaling mechanisms or if there are other modes of communication such as nerve‐to‐cancer cell synapses, electrical coupling, or synapse‐like structures occurring outside of the CNS. Furthermore, the functions of various peripheral glial cells in the connections between nerves and cancer cells outside of the CNS have not been well investigated.

Then in the following text, we further elucidate the precise mechanisms of nervous system–cancer interactions, focusing on defining and therapeutically targeting nervous system–cancer communications, both in the CNS, as well as PNS local TME and systemically.

## 3 NERVOUS SYSTEM REGULATE BRAIN CANCERS INITIATION AND PROGRESSION

It is on this backdrop of neuronal activity‐regulated brain development and plasticity that we have to consider the interactions of neurons with various brain cancers. The most common primary brain cancers are gliomas, and most currently published studies mainly focus on glioma; thus, in current review, the focus will be on gliomas. Gliomas extensively infiltrate the brain and spinal cord; however, peripheral progression is rare. GBM growth is modulated by intracellular mechanisms and crucial microenvironmental networks.^7^ Neurons are an essential part of the GBM microenvironment and modulate malignancy. The release of electrochemical and activity‐regulated growth factors^8^, ^18^ mediate this microenvironmental communication. The neural‐GBM crosstalk provides efficient treatment targets, such as activity‐induced growth factors release,^8^, ^18^ ion channel activity, neuron‐to‐GBM neurotransmission, and gap junction association. Therefore, regulating GBM's impact on neuronal excitability furnishes an opportunity to reduce activity‐regulated GBM progression. Currently, much research has discovered that mechanisms such as synaptic neurotransmission, activity‐dependent neuronal signaling, and regulatory circuits^4^ promote GBM growth and have illustrated the undetermined potential to focus these mechanisms on treating lethal cancers.

### 3.1 Direct nervous system–cancers interactions

Tumorigenesis involves acquiring new functions (hallmarks of cancer) necessary for malignancy.^4^ Recently, different provisional hallmark parameters, such as nonmutational epigenetic reprogramming, phenotypic plasticity, polymorphic microbiomes, and senescent cells in the TME, have stimulated debate and experimental elucidations.^5^ The intersection observed between the nervous system and cancers, including tumors’ systemic effects on nervous system function (e.g., cachexia, sleep disruptions, cognitive impairment, etc.), tumor‐induced local tissue innervation remodeling, and nervous system’ modulatory influence on tumor phenotypes.^19^, ^20^, ^21^, ^22^, ^23^ However, the cellular and molecular level interconnections of nervous system with developing cancers allow the acquisition of hallmark function, which has not been assessed yet.

**FIGURE 2:**
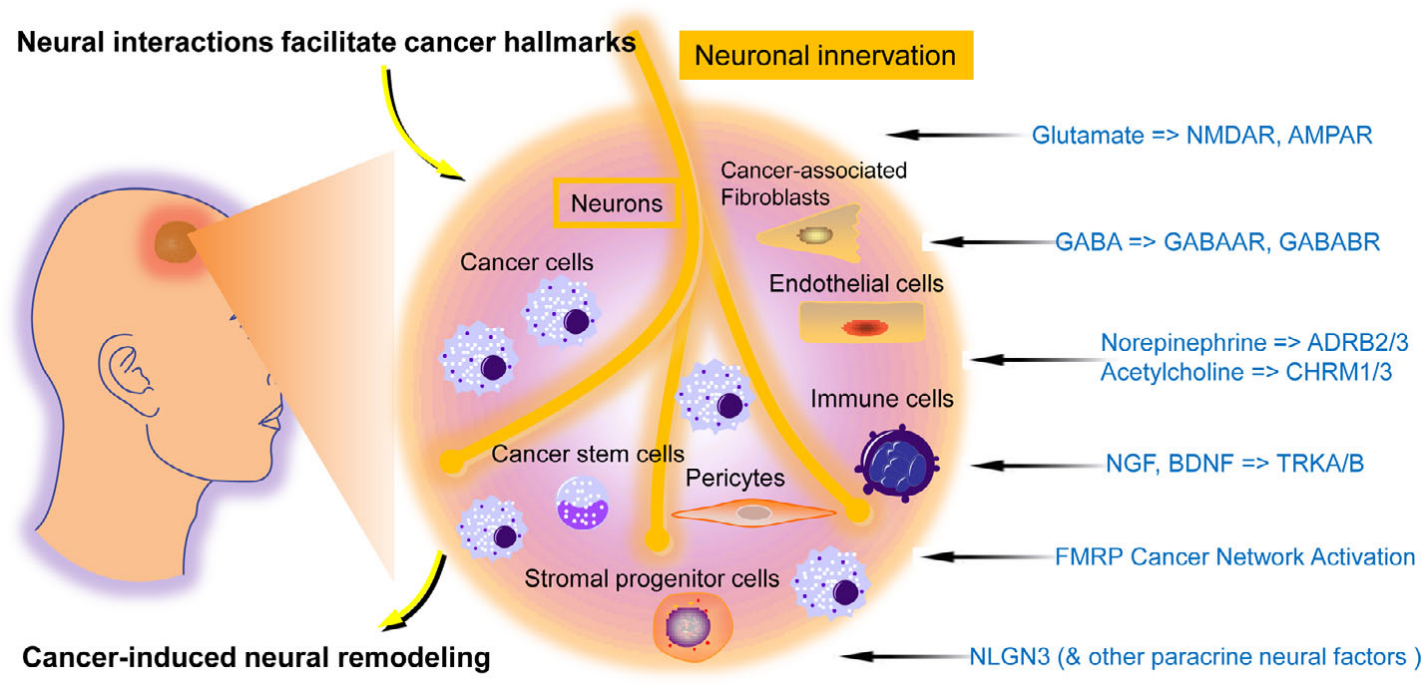
Neurons, their innervation, signaling, and regulatory networks could induce cancer hallmarks. First, neuronal innervation (axonal projections) has three primary subtypes: sensory, motor, and autonomic nerves. Signaling between axonal projections and cancer cells induces various cancer hallmarks, while reciprocal cancer cells’ effects on the nervous system result in neural form and role remodeling, causing neurological cancer complications and enhancing neuronal consequences in cancer pathophysiology. Second, neuronal regulatory pathways co‐opted in cancer cells also facilitate hallmark capabilities acquisition, as evident by the increasing breadth of co‐opted neuronal regulatory circuits in cancer cells. NMDAR, N‐methyl‐d‐aspartate‐receptor; AMPAR, α‐amino‐3‐hydroxy‐5‐methyl‐4‐isoxazole‐propionic acid receptor; GABA, γ‐aminobutyric acid; GABAAR, γ⁃aminobutyric acid type A receptor; GABABR, γ⁃aminobutyric acid type B receptor; ADRB2/3, adrenergic receptor beta 2/3; CHRM1/3, muscarinic acetylcholine M1 receptor 1/3; NGF, nerve growth factor; BDNF, brain‐derived neurotrophic factor; TRKA/B, tyrosine kinase receptor A/B; FMRP, fragile X mental retardation protein; NLGN3, neuroligin‐3.

The hallmark functions are greatly affected by neurons and innervation.^4^ The nervous system arborizes throughout the body, regulating movement and sensation, and innervates tissue stem cell niches to modulate the homeostasis, growth, and regeneration of various tissues and organs. Similarly, nervous system also regulates cancer phenotypes, often by neural mechanisms co‐option that functions parallelly in healthy tissues. The literature has indicated how neuronal innervation and neuronal projections (axons) affect the acquisition of different hallmark functions (Figure 2). Generally, neuronal activity stimulates proliferative signaling, conveys cell death resistance, promotes invasion and metastasis, and stimulates inflammation induced by tumors.^4^ The neurons had a striking influence on GBM development and proliferation,^7^, ^18^ promoting a beyond neuron‐regulated paracrine mitogens search for further mechanisms, which indicated functional synaptic signaling of GBM cells with glutamatergic neurons via calcium‐permeable AMPA receptors causing depolarizing currents in the cancer cells in both pediatric and adult GBM.^9^ This synaptic interaction modulates GBM cell development and proliferation, as seen with genetic inhibition (dominant‐negative GluA2 subunit expression of AMPA in GBM cells) or by pharmacological AMPA receptors suppression in GBM‐neuron coculture and in vivo.^7^ A second neuron‐to‐GBM synapse type has been determined, comprising GABAergic interneurons and GBM cells with GABAA receptors.^24^ The electrochemical current after synaptic signaling essentially promotes proliferation: only membrane depolarization is sufficient for GBM proliferation.^7^ This process depicts neural signaling that induces the possession of essential cancer‐proliferative signaling hallmarks. The neuronal synaptic and paracrine activity‐induced GBM growth act as mitogens; NLGN3 and BDNF stimulate neuron‐to‐GBM synaptogenesis.^7^, ^25^ Therefore, brain TME neuronal activity promotes sustaining proliferative signaling hallmark by paracrine signaling that triggers oncogenic mechanisms and neuron‐to‐cancer synaptic signaling, a canonically neuronal mechanism. NLGN3, neuron‐mediated growth molecules for optic and other GBMs, is essential for optic nerve‐modulated tumorigenesis as its genetic ablation phenocopies the dark rearing (limiting visual experience) effect on tumor initiation.^26^ Interestingly, reducing the optic nerve function by dark rearing after the temporal window of tumor initiation also notably reduced tumor quantity, highlighting its tumor maintenance role. This can only be achieved via tumor suppression, linking optic nerve function with tumor maintenance via cell death‐resisting hallmark^26^; further research will assess the type of programmed cell death in suppressed tumors. These data demonstrated the importance of innervation for cell death‐resisting hallmarks in different tumors. Furthermore, TME neurons secrete a paracrine factor midkine, which recruits and activates CD8^+^ lymphocytes to release CCL4 chemokine, promoting CCL5 expression in microglial/myeloid cells, which in turns stimulates the cell cycle (proliferative signaling) and inhibits apoptosis in cancer cells.^27^ It is remarkable how this mechanism conveys the hallmark ability to hide from the immune system by CD8^+^ T cells, without impeding their infiltration and chemo‐attraction, but by controlling the recruited ostensibly ‘‘activated’’ CD8^+^ T cells, without T cell attack and GBM cells killing. CD8^+^ T cells allow a unique type of tumor‐induced inflammation that promote the hallmark functions of resisting cell death, sustaining proliferative signaling, and evading immune destruction.^27^ These data suggest that tumor innervation is an essential hallmark‐facilitating TME factor (Figure 2).

### 3.2 Paracrine/autocrine interactions

Another aspect of the neural and cancer interaction is linked with neuronal signaling levels and modulatory cancer cell circuits of different origins and not only with neural–cancer links.^4^ Cancer cells express different signaling receptors stimulated by their cognate ligands’ autocrine and paracrine supply; the latter often comprises “feedforward” ligand interchanges with different innervation subtypes. Together with classically neuronal signaling mechanisms considered here, cancer cells exhibit distinct neuronal structural characteristics, including long neurite‐like processes extension that promote cell‐to‐cell interactions in TME.^28^, ^29^ Various research has indicated that different aberrant co‐opted neuronal modulatory mechanisms in cancer cells influence the functional activities that promote cancer pathogenesis (Figure 2), suggesting that besides neuronal innervations and axons, cancer cells’ co‐option of neuronal modulatory circuits also orchestrate hallmark capabilities.^4^

First, neurotransmitters and neurotrophins‐mediated paracrine/autocrine signals stimulate growth and vascularization. Acetylcholine (ACh) also induced nerve growth factor (NGF) in a mouse gastric cancer model by stimulating its receptor‐muscarinic receptor‐3 (CHRM3), causing autocrine tumor advancements and paracrine increase of cholinergic hyper‐innervation.^13^ Here, ACh release was controlled by cholinergic neurons and intestinal tuft cells, which promoted CHRM3 signaling to stimulate NGF expression and release, causing cancer cells’ autocrine TRK receptor signaling. Paracrine‐induced increase of ACh‐expressing tuft cells and the ingrowth and cholinergic nerves increment in TME. Together, these communications enhanced tumor‐inducing signals, such as WNT and YAP pathways activation, known to increase cancer growth.^4^ In this model, the NGF and neurotransmitters collectively influenced the proliferative hallmark; potential influence on other hallmarks was not studied. Another study suggested that noradrenaline signaling via ADRB2 is another hallmark function: stimulating angiogenic switch that mediates and maintain tumor vascularization for tumor progression.^10^ Neurotrophins and neurotransmitter signaling pathways are also essential in CNS cancers; for instance, in GBMs, AMPA receptors mediated glutamatergic signaling,^9^, ^30^ and NMDA receptors in breast cancer metastasized to the brain.^21^ In GBM, such neurotransmitter signals are elaborated and augmented by neurotrophin (BDNF) signals that enhance the numbers and strength of glutamatergic neuron‐to‐GBM synapses.^25^

Second, GABA also modulates autocrine proliferation and immune evasion signaling. GABA is a CNS inhibitory neurotransmitter produced after converting glutamic acid decarboxylases (GAD1/2) into intracellular glutamine and acts via GABAAR and GABABR receptors.^4^ GABA levels are enhanced in the late‐cancer stage and are inversely linked with prognosis. The levels of GAD1 and GABABR are also inversely correlated; these are coexpressed in cancer cells and generate an autocrine signaling loop.^31^ Pharmacological and genetic GAD1 and GABABR perturbation in mouse tumor models and cell lineages has indicated that GABA‐regulated signaling promotes the hallmark functions of sustaining proliferation and evading immune destruction.

Third, glutamate could mediate paracrine/autocrine signals of the metastatic/invasive hallmark. Another crucial co‐opted neuronal signaling pathway is the glutamate–NMDA receptor signaling, linked with synaptic transmission. Even though paracrine synapses were induced in pancreatic tumors by neuroendocrine and ductal‐autocrine signaling.^32^ Upregulated glutamate transporters secrete glutamate and stimulate NMDAR in the same tumor cells, influencing two hallmarks: proliferation and, more importantly, invasion.^32^, ^33^ As discussed, glutamatergic signals via synapses promote tumor invasion and GBM brain colonization.^30^ In neurons, FMRP is an essential downstream factor of glutamate‐induced NMDAR signaling; it modulates metastasis, invasion, and immunosuppression. The cancer cells’ co‐option of neuronal modulatory mechanisms includes more than ligand–receptor signaling, such as FMRP.^4^ It is an RNA‐binding protein responsible for mediating messenger RNA (mRNA) stability and protein translation, influencing the expression and function of multiple genes. It is widely overly expressed in solid human tumors, many lacking NMDAR function, suggestive of additional modulatory pathways.^34^ Initially, FMRP was used as a metastasis and invasion regulator.^33^ Recently, it was revealed that it is a master immunosuppressive TME modulator in different tumor models and is linked with human cancers.^34^ Therefore, FMRP expression and the regulatory genes in cancer cells are linked with the hallmarks mediating morbidity and death, metastasis and invasion, and immune escape. These studies highlight a remarkable feature of different cancer cells, such as activating several neuronal regulatory pathways promoting tumor initiation and progression (Figure 2).

Overall, the multifactorial neurotrophins and neurotransmitters amplification linked with paracrine and autocrine signaling interaction between neurites and cancer cells in TME promote the acquisition of different hallmark abilities.

## 4 BRAIN CANCERS INFLUENCE THE FUNCTION OF THE CNS

Just as the nervous system can regulate cancer progression, cancer also remodels neuronal excitability and thus neuronal activity.^35^, ^36^, ^37^ Interactions between the nervous system and cancer occur both in the local TME and systemically.^35^ Elucidating the precise mechanisms operant in the tumor or TME of each molecularly defined malignancy is of great importance for choosing helpful medications. In current review, the focus will be on gliomas, since the most common primary brain cancers are gliomas, and most currently published studies also mainly focus on glioma.

### 4.1 Gliomas innervate neural circuits via neural network integration (including synapses and gap junctions)

**FIGURE 3:**
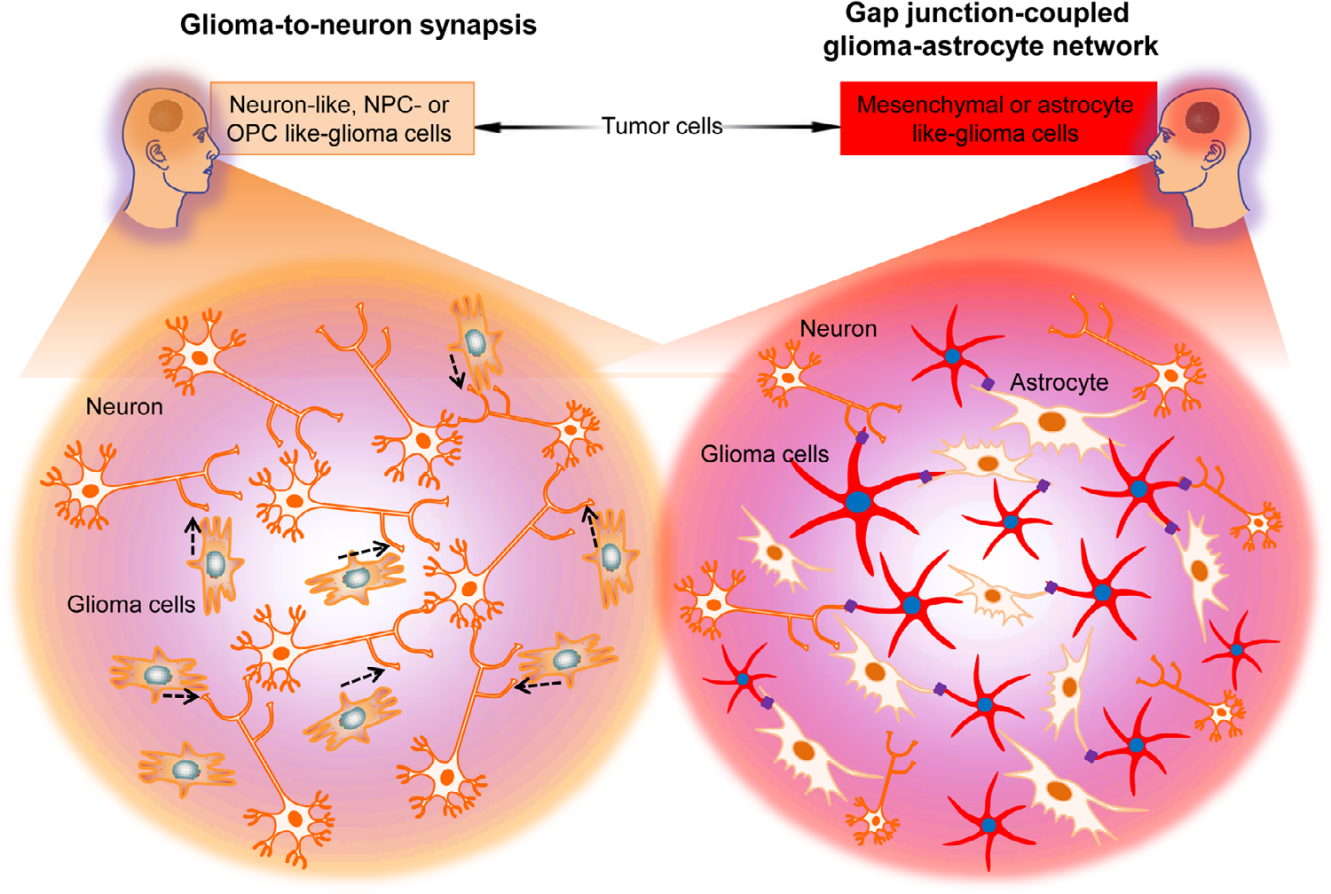
CNS cancer cells influence neural networks by generating invasive stationary glioblastoma networks. Distinct glioblastoma cell subpopulations invade astrocytic and neuronal networks. First, neural precursor cells (NPC)‐like, oligodendrocyte precursor cells (OPC)‐like, or neuron‐like tumor cells form glutamatergic‐neuronal synapses, driving neural circuits’ GBM invasion via the calcium‐dependent pathway. Second, a stationary astrocyte (AC)‐like or mesenchymal (MES)‐like tumor cell network forms functional connections through gap junctions with each other, normal astrocytes, and neuronal input. Glioblastoma cells transition to a gap junction‐coupled network occur over time; however, both states stimulate tumor growth.

Gliomas invade neural circuits via glutamatergic (calcium‐permeable AMPAR regulated) neuron‐to‐GBM synapses.^7^, ^30^ Invaded circuits’ neuronal activity triggers GBM progression via paracrine mitogenic factors^37^ and electrochemical signaling. Neuronal activity induces electrochemical signaling, depolarizing the GBM cell membrane and promoting tumor development. However, the voltage‐dependent pathways are still undetermined.^7^ The GBM cells subset that responds to neuronal signals with inward, slow, potassium‐induced currents also forms GBM‐to‐GBM networks by coupling gap junctions^7^, ^30^ between tumor microtubes (long cancer cell processes)^29^ (Figure 3). This GBM‐coupled gap‐junction network, reminiscent of AC‐coupled gap‐junction network present in the healthy brain, promotes these electrochemical signals and the subsequent calcium transients via the GBM network,^7^, ^30^ enhancing potassium‐stimulated currents^7^ and therapeutic resistance.^29^ However, the mechanisms modulating the establishment of the GBM circuit, its expansion, and evolution during the disease course, how the disease so widely affects the brain, and which cellular states are linked with pathogenic malignant network formation require further research.

Venkataramani et al.^30^ used advanced techniques to elucidate how the normal neural circuits are invaded by the GBM network and demonstrated that distinct glioblastoma cells subpopulations form different intercellular links with neural cells in the TME and structurally invade the neural networks in certain ways. Dynamic, invasive glioblastoma cells in states resembling NPCs [NPC‐like or oligodendrocyte precursor cell (OPC)‐like cells] that form synapses with neurons but do not connect other GBM cells with gap junctions. However, stationary gap junction‐coupled cells primarily in mesenchymal (MES)‐like or AC‐like cells states are associated with coupled gap junctions, interconnected tumor networks connected to normal ACs through gap junctions^30^ (Figure 3). Therefore, neuronal circuits are invaded by invasive NPC/OPC‐like GBM cells synaptically, and AC/MES‐like GBM cells coupled with gap junction integrate via gap junctions into astrocytic networks, in addition to receiving input from neurons.

The NPC/OPC‐like invasive GBM cells respond to neuronal stimuli by elongating tumor microtubes to invade healthy brain tissue and by accelerating cellular invasion speed,^30^ like the migration pattern observed in normal NPCs and OPCs. Therefore, during cortical development, glioblastoma cells invade the transient synapses form on migrating neuroblasts.^30^ The calcium transients induced by synaptic interactions^7^, ^9^ are crucial for this invasion. The research further indicated that calcium signaling causes tumor cell invasion and is inhibited by calcium chelators or CREB suppression,^30^ indicating that the calcium transients can experimentally modulate electrochemical interaction in GBM and are the primary component involved in the membrane depolarization mechanism that promotes tumor progression. In the stationary GBM‐to‐GBM‐to‐AC network, connectivity enhances with time, predicting enhanced resistance to treatment^29^ with tumor adaptation,^30^ consistent with clinical data. These researches indicate advances in glioblastoma development and treatment resistance throughout the disease. It is, however, clear that GBMs neuroscience will help understand and, ultimately, provide effective therapy against highly mortal brain cancers.

### 4.2 Glioma remodeling of human neural circuits as the way GBMs decrease survival

Gliomas synaptically unite with neural circuits,^7^, ^9^ and there is bidirectional communication between neurons and GBM cells, where neuronal activity promotes GBM progression,^7^, ^9^, ^18^ and GBMs enhance neuronal excitability.^38^, ^39^, ^40^, ^41^ Glioblastomas induce neuronal hyperexcitability by releasing nonsynaptic glutamate and synaptogenic components^35^, ^36^ and suppressing inhibitory interneurons.^40^ In awake, resting patients, it has been indicated that the glioblastoma‐infiltrated cortex had enhanced neuronal excitability.^7^ The mechanisms of glioblastomas that maintain their engagement with neuronal networks and dysregulate cortical function still need elucidation and may highlight therapeutic targets for these brain cancers.

Krishna et al.^42^ carried out intraoperative electrophysiology analysis while the patients were engaged in language tasks, assessed glioblastoma‐infiltrated cortexs’ local field potentials during speech initiation, identified neural responses decodability, and indicated glioblastoma cells’ synaptic enrichment stimulators. Briefly, they performed a short‐range electrocorticography assessment of the tumor‐infiltrated cortex to indicate language‐task‐specific functional remodeling and activation of language circuits. Furthermore, it was revealed that specific intratumoral regions maintained functional connectivity by thrombospondin/thrombin sensitive protein 1 (TSP‐1)‐expressing malignant cells subpopulation (high functional connectivity (HFC) GBM cells), suggesting that pharmacological suppression of TSP‐1 reduces glioblastoma cell growth and network synchrony in TME and highlighting an efficient therapeutic strategy for future clinical research.^42^ Moreover, it was determined how GBM‐mediated neuronal alterations affected cognition‐associated neural circuits and if these interactions impacted individuals’ survival. Additionally, it was noted that the extent of functional connectivity between a healthy brain and glioblastoma negatively affects individuals’ survival and language task outcomes. This investigation revealed that high‐grade GBMs functionally alter neural networks in the human brain, inducing tumor growth and cognition impairment.^42^

How GBM‐network communications affect neuronal activity and cognition in patients remains undetermined. The neuronal microenvironment is a critical modulator of GBM progression. Both connectivity remodeling and paracrine signaling might influence network‐level alterations in patients, influencing cognition and survival. Some studies with a heterogeneous population of patients indicated that the overall survival was increased with functional connectivity^43^, ^44^, ^45^; however, these data are largely affected by limited spatial resolution, tumor vascularity, and a heterogenous patient cohort. Nonetheless, Krishna et al.^42^ need to address the direction of causality. It is still possible that functionally connected cortical regions that originated GBM are more significantly connected and, therefore, may have larger network distribution, thereby supporting distinct migratory glioblastoma subpopulations.^30^ A better understanding of neurons and GBMs cross‐talk and how functional integration influences clinical results will allow more pharmacological and neuromodulation treatment options to improve cognitive and survival outcomes.

## 5 INTERACTIONS BETWEEN THE PNS AND CANCERS

The PNS is responsible for innervating many organs and tissues in the body, encompassing autonomic, motor, and sensory components. The autonomic PNS is responsible for regulating both adrenergic (sympathetic) and cholinergic (parasympathetic) autonomic responses. These responses play a crucial role in facilitating muscle contractions and gland secretions, which are essential processes for maintaining the appropriate functioning of visceral organs.^46^ Peripheral nerves play a vital role in the stem cell compartments as evident in many organ systems and tissue types.^47^, ^48^, ^49^, ^50^, ^51^, ^52^ Significant involvement of peripheral innervation in the advancement of several types of malignancies, such as breast, prostate, gastric, and pancreatic cancers, paralleling the regulatory functions of the PNS in homeostasis, development of the tissue, and regeneration.

### 5.1 Breast cancer

Several studies have demonstrated the importance of the PNS in the initiation and progression of breast cancer, with particular attention to the existence of adrenergic sympathetic innervation in the breast tissues.^53^, ^54^, ^55^ The examination of breast cancer samples obtained from individuals has revealed a significant association between perineural invasion and the advancement of the disease, metastasis, and the determination of the disease's clinical stage.^56^ Previous in vitro experiments have indicated that the cocultivation of human breast cancer cells with rat neurons led to an elevation in the synthesis of NGF by the breast cancer cells. This, in turn, resulted in an augmented proliferation of neurites by the surrounding nerves.^57^ Additional in vivo investigations have revealed the presence of sympathetic innervation in breast tumors in human subjects, as well as in preclinical mice models that exhibit spontaneous mammary tumors.^58^ The targeted activation of sympathetic nerve fibers inside a xenograft of an orthotopic human breast cancer model was shown to facilitate the primary breast tumor growth and the development of distant metastasis. This effect was attributed to the local release of noradrenaline. In these breast cancer models, the application of chemical ablation on the sympathetic nerve fibers has shown a decrease in immunosuppressive processes. This reduction is characterized by a decrease in the number of regulatory T‐cells (T_regs_) that infiltrate the tumor and a decrease in the generation of immunological checkpoint signals, such as programmed cell death protein (PD)‐1 and PD‐L1. In comparison with the sympathetic nerves present locally, the activation of parasympathetic nerve fibers within the microenvironment of breast cancer has been observed to lead to a reduction in the growth and the occurrence of distant metastasis of the tumors. Additionally, this stimulation has been found to decrease the production of immunological checkpoint markers. Similar to the function of parasympathetic nerves, the augmentation of metastases and the stimulation of a more aggressive breast cancer phenotype were seen with the elimination of sensory neurons via treatment with high‐dose capsaicin.^59^ In summary, the outcomes indicate that although parasympathetic and sensory nerve processes have a suppressive influence on breast cancer growth, sympathetic nerves may contribute to the development of breast cancer. These findings underscore the significance of nervous system control in shaping the immunological microenvironment of tumors.

### 5.2 Prostate cancer

The prostate is an organ that possesses a high density of nerve fibers, and its development, homeostasis, and function are regulated by both parasympathetic and sympathetic signals. Histopathological examinations of prostate cancer have revealed the presence of perineural invasion, a condition characterized by the infiltration and proliferation of tumor cells along nerve structures.^60^, ^61^ Significant initial in vitro investigations were conducted utilizing a coculture system including a dorsal root ganglion and human prostate cancer cells. This research showed that neurons had the ability to enhance the proliferation of prostate cancer cells, hence offering one of the earliest indications of a growth‐stimulating influence exerted by neurons on cancerous cells.^62^ Research by Frenette and colleagues provided experimental evidence that both sympathetic and parasympathetic nerves drive prostate cancer.^16^ Orthotopic prostate cancer xenografts generated from patients were efficiently prevented from developing when adrenergic (sympathetic) nerves in the mouse prostate were chemically or surgically ablated. Moreover, the development of these orthotopic prostate cancer xenografts was significantly reduced in recipient mice by the genetic ablation of β2‐ and β3‐adrenergic receptors. The development of prostate cancer is also influenced by parasympathetic nerves. According to the study, prostate cancer cells that were orthotopically transplanted may spread and metastasize more easily if muscarinic ACh receptor‐1 (Chrm1) signaling is activated inside the tumor stroma. The collective results of these pioneering insights by the Frenette research group have shown the essential and intricate interplay between prostate cancer and the neurological system.^16^

### 5.3 Pancreatic cancer

Studies regarding the histological examinations of human pancreatic cancer specimens revealed a notable presence of perineural invasion in pancreatic ductal adenocarcinoma (PDAC).^63^, ^64^, ^65^ The initial correlative investigations conducted on human patients also showed a connection between the expression of NGF and perineural invasion. The research found that NGF expression was associated with the course and prognosis of pancreatic cancer.^66^, ^67^ Recent research has elucidated the essential molecular pathways that enable the malignant intercommunication between the pancreas and nervous system in the context of cancer. The roles of the parasympathetic and sympathetic nerve systems in pancreatic cancer have been adequately shown by various research. The progression of PDAC has been associated with catecholamines that are generated by the sympathetic nervous system. In reaction to stress, these catecholamines might be produced systemically by the adrenal glands or locally by neurons.^68^, ^69^, ^70^ The study demonstrated that catecholamines had a direct impact on promoting the release of NGF from PDAC cells. This, in turn, results in enhanced neurite outgrowth, increased density of nerves, and expedited development of tumors. In comparison with adrenergic sympathetic signaling, cholinergic parasympathetic signaling tends to inhibit the development of pancreatic tumors. Studies using transgenic mouse models of PDAC have demonstrated that parasympathetic denervation (vagotomy) increases the risk of pancreatic carcinogenesis. This increase was attributed to the proliferation of cancerous CD44+ epithelial cells within the tumor stroma.^71^ In general, the transmission of signals from neurons to cancer cells facilitates the advancement of the disease since cancer cells actively modify the nervous system to amplify the protumorigenic signals originating from neurons.

### 5.4 Gastrointestinal tract cancers

**FIGURE 4:**
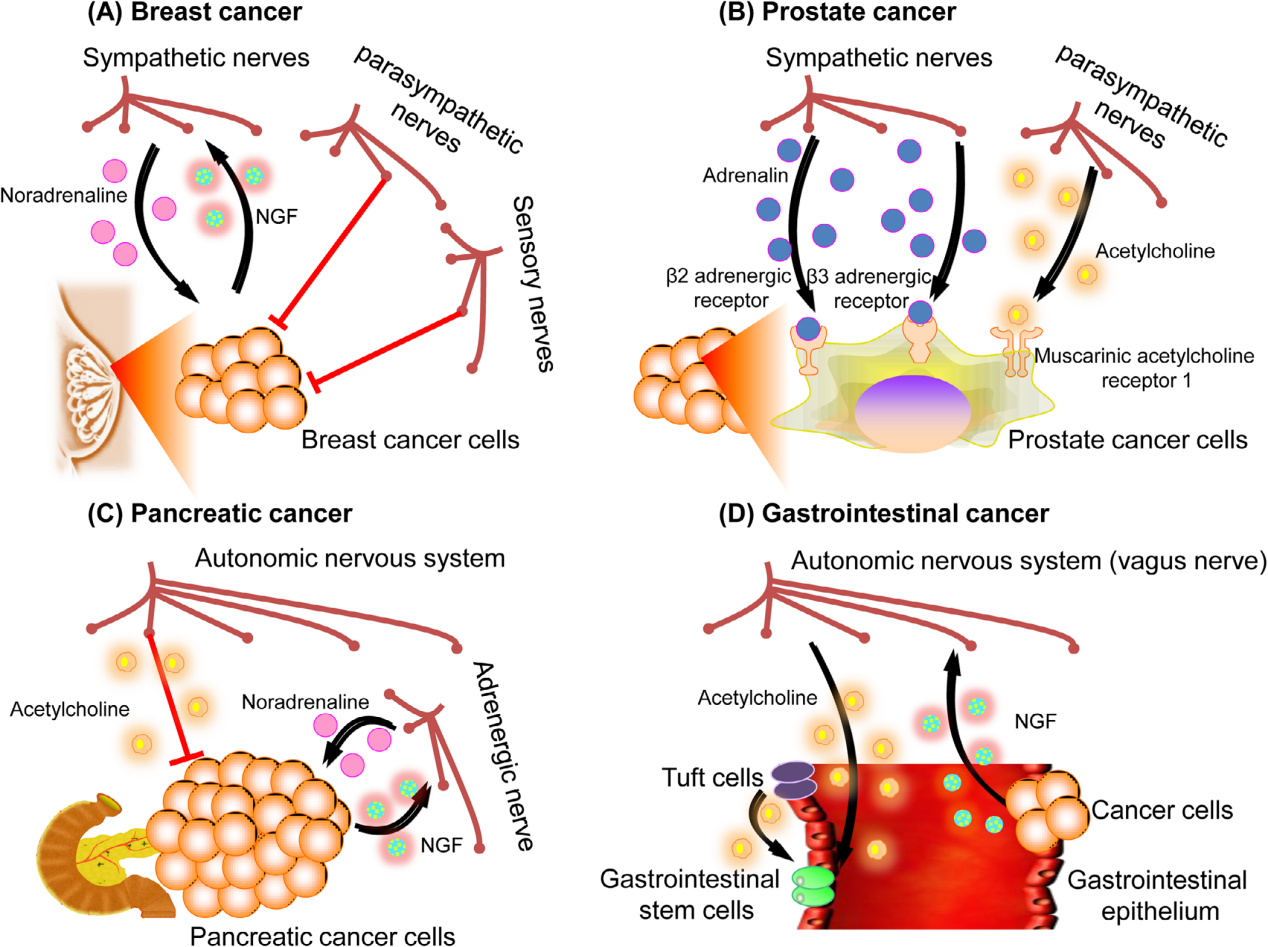
Peripheral nervous system regulation of cancer. (A) Nerve–cancer interactions in breast cancer. In breast cancer, specific stimulation of local sympathetic nerve fibers can promote breast cancer growth through locally released noradrenaline. And the increased nerve growth factor (NGF) production by cancer cells could cause enhanced neurite outgrowth. However, stimulation of parasympathetic nerve fibers and sensory neurons in the breast cancer TME can decrease tumor growth and metastasis. (B) Nerve–cancer interactions in prostate cancer. In prostate cancer, adrenergic (sympathetic) nerves could promote cancer progression via β2‐ and β3‐adrenergic receptors. Parasympathetic nerves also influence prostate cancer progression via muscarinic acetylcholine receptor 1 (Chrm1) signaling. (C) Nerve–cancer interactions in pancreatic cancer. In pancreatic cancer, acetylcholine can suppress pancreatic tumorigenesis. By contrast, β‐adrenergic signaling (noradrenaline) promotes pancreatic cancer growth, and pancreatic cancer cells secrete NGF to increase sympathetic innervation of the TME. (D) Nerve–cancer interactions in gastrointestinal cancers. In gastrointestinal cancer, acetylcholine signals to cancer cell muscarinic acetylcholine receptors to promote cancer growth, while cancer cells secrete axonogenic factors such as NGF to increase nerve ingrowth in the TME.

Previous studies have shown that human and animal models of colon cancer have increased nerve ingrowths.^72^ The correlation between overall survival and nerve density in human patients supports this conclusion.^73^ In a rat model of gastric cancer caused by exposure to carcinogens, the use of vagotomy for the stomach led to a reduction in the overall number and size of gastric tumors.^74^ Prior research has elucidated the underlying processes that contribute to the promotion of tumor growth through innervation in a spontaneous gastric malignancy of mice model.^74^ The crucial function of cholinergic innervation in gastric tumor initiation and development was clearly shown by surgical or pharmacological cholinergic denervation during the preneoplastic stage of gastric carcinogenesis.^74^ The activation of cholinergic neuronal transmission in gastrointestinal cancer stem cells leads to an increased activity of the downstream Wnt signaling pathway, hence facilitating the process of carcinogenesis. This effect is mediated through the interaction with Chrm3. The gastrointestinal epithelial tuft cell subtype serves as the primary origin of ACh, which promotes tumorigenesis inside the stomach mucosa.^13^ The tuft cells are responsible for synthesizing ACh, which in turn stimulates the synthesis of NGF in gastric stem cells. This process facilitates the development of axons (axonogenesis) and promotes the proliferation of cholinergic nerves inside the TME, resulting in heightened release of ACh from these neurons. The administration of ACh has been found to enhance the production of NGF in mouse models of gastric carcinoma.^13^ Notably, the upregulation of NGF expression, resulting in elevated levels of ACh inside the gastric stem cell niche, has been demonstrated to be adequate for the initiation of tumorigenesis.^13^ The significance of cholinergic signaling in gastrointestinal malignancies differs significantly from its role in PDAC, highlighting the suggestion that while the nervous system consistently plays a crucial role in regulating cancer, the involvement of specific nerve types or neurotransmitters can vary across different types of cancers (Figure 4).^75^

## 6 EXOSOMES MEDIATE “WIRELESS COMMUNICATIONS” BETWEEN THE NERVOUS SYSTEM AND CANCER

The initial identification of exosomes dates back about four decades, during that time they were mostly regarded as a biological mechanism for waste elimination.^76^, ^77^ Nevertheless, recent significant findings provide evidence supporting their involvement as active mediators in cellular communication. Numerous bioactive compounds, including mRNA and microRNAs, are present in exosomes. These molecules are then taken in by the cells.^78^ The human brain has a diverse array of non‐neuronal cells, such as oligodendrocytes, microglia, ACs, and normal fibroblasts (NFs). It is well acknowledged that tiny extracellular vesicles (EVs), particularly exosomes released by these non‐neuronal cells, exert an influence on several neuronal processes.^79^ Significant progress has been achieved in the field of neuronal exosome research since Sadoul's group initially demonstrated the secretion of exosomes by postmitotic neurons.^80^, ^81^, ^82^ Presently, there is a widely accepted understanding that central neurons possess the capability to both release and internalize tiny EVs, particularly exosomes. One particular kind of miniature EV that comes from intraluminal vesicles inside multivesicular structures is called an exosome. Numerous neuronal functions, including mRNA expression, synaptic plasticity, axon guidance, neurogenesis, synapse elimination, inflammation, neuroprotection, and synaptogenesis, have been shown to be impacted by specific compounds carried by neuronal exosomes.^79^, ^83^, ^84^, ^85^, ^86^, ^87^, ^88^, ^89^ Hence, it is widely believed that the transmission of exosomes, facilitating volume transmission, plays a significant role not only in the modulation of neuronal function based on activity but also in the preservation and regulation of local circuitry homeostasis.^79^ Only the most recent research on the signaling function of neural and non‐neuronal exosomes in the context of cancer neurology will be included in this review.

### 6.1 Nervous system exosomes exert big roles in cancer progression

It has been widely recognized that in addition to neurons and numerous non‐neuronal cells, cancer cells are capable of secreting exosomes.^90^ Exosomes can be found in several biofluids, including cerebrospinal fluid (CSF), urine, and blood.^91^ In addition to exosomes, additional EVs commonly encompass microvesicles and apoptotic bodies, which exhibit distinct characteristics in terms of their biogenesis and expression of molecular markers.^92^, ^93^ Exosomes are now recognized for their significance in intercellular communication and their pivotal role in several physiological and pathological processes, including cancer.^94^ In the context of cancer, these factors have a role in stimulating angiogenesis, facilitating cell proliferation and migration, inflammatory response elicitation, suppressing the immune system, evading immune surveillance, and promoting metastatic spread.^95^, ^96^ The cargo found within nervous system exosomes includes a variety of components, including enzymes, lipids, DNA fragments, mRNAs, proteins, transcription factors, long noncoding RNAs (lncRNAs), and micro RNAs (miRNAs).^97^, ^98^, ^99^ The molecules can be transferred into cells of stromal origin to facilitate cross‐talk within the TME. Thus, allowing the cells to become tumorigenic and enhance the growth of the primary malignancy by altering the phenotype of the cells receiving them.^94^, ^95^, ^96^, ^100^, ^101^

The TME is a crucial factor in the progression of primary tumors and the spread of cancer cells to other parts of the body since it facilitates effective communication involving cancer cells with surrounding or distant cells. The TME has several components, including the extracellular matrix (ECM), endothelial cells, cancer‐associated fibroblasts (CAFs), immunological cells, and MES stem cells (MSCs).^102^, ^103^, ^104^, ^105^ It has been established that exosomes originating from primary tumor cells possess the ability to initiate the conversion of fibroblasts into myofibroblasts. These myofibroblasts are capable of secreting matrix metalloproteinases, which subsequently break down the ECM. This degradation process results in the liberation of several chemicals that facilitate the invasion of cancer cells to neighboring tissues. Furthermore, it has been observed that the exosomes originating from the nervous system have the ability to induce the development of fresh blood vessels (angiogenesis) by activating macrophages inside the TME, therefore establishing an inflammatory habitat. Exosomes possess the capability to trigger epithelial‐to‐MES transition (EMT), a process characterized by the loss of cell–cell adhesion and detachment of epithelial cells from the tumor. This phenomenon facilitates the spread of cancer cells, which is considered a key feature of metastasis.^106^, ^107^, ^108^ There has been a suggestion that exosomes released by MSC‐differentiated adipocytes have a role in promoting EMT in breast cancer cells via activating the Hippo signaling pathway. The confirmation of this was shown by the phosphorylation of two important transcription factors within this pathway, namely YAP and TAZ.^109^ Previous research has demonstrated that exosomes originating from lung cancer cells with a high propensity for metastasis, as well as exosomes derived from the serum of individuals in the advanced stages of lung cancer, elicit the migration, invasion, and proliferation of human bronchial epithelial cells. Additionally, these exosomes induce upregulation of vimentin, a marker that is closely linked to EMT and metastasis.^110^

Tumor cells and the TME are subjected to several factors, including hypoxia and acidity. Conversely, the acidity of the microenvironment can also impact the interaction between tumor cells and adjacent cells in the TME.^90^ However, tumor cells easily adapt to these adverse conditions by modifying their surrounding microenvironment, hence facilitating the advancement and spread of the tumor. The cancer cells harness the production of exosomes as a strategy to remodel the microenvironment and adapt to hypoxia. Bladder cancer cells, when subjected to hypoxia, exhibit the release of exosomes that contain elevated quantities of lncRNA–UCA1. This phenomenon subsequently facilitates EMT and the advancement of cancer.^111^ In addition to affecting cancer cells, hypoxia also affects adjacent stromal cells. Research conducted on lung cancer has shown evidence that exosomes, which are released by bone marrow‐derived MES stem cells (BMSCs) experiencing hypoxia in the TME, play an essential part in facilitating the propagation and invasion of cancer cells as well as triggering EMT. This process is mediated by the transfer of certain microRNAs (miR‐193a‐3p, miR‐210‐3p, and miR‐5100) from hypoxic BMSCs to cancer cells.^112^ Additionally, the presence of an acidic microenvironment can be attributed to the upregulation of glycolysis and subsequent lactate generation, which leads to a reduction in pH. Research conducted on melanoma has shown evidence that the presence of an acidic microenvironment significantly increases the release of exosomes during the metastatic noninvasive phase. This heightened exosome release facilitates the spread and infiltration of cancer cells to other organs of the body by facilitating the transfer of substances that promote the formation of metastases. Therefore, it can be inferred that the quantity of exosomes released by cancer cells likely serves as an indicator of the progression of the disease.^113^ Importantly, previous research has demonstrated that some components of the TME, like the presence of MSCs and CAFs, are capable of releasing exosomes that facilitate the reprogramming of adjacent cells and contribute to the progression of cancer.^90^

**FIGURE 5:**
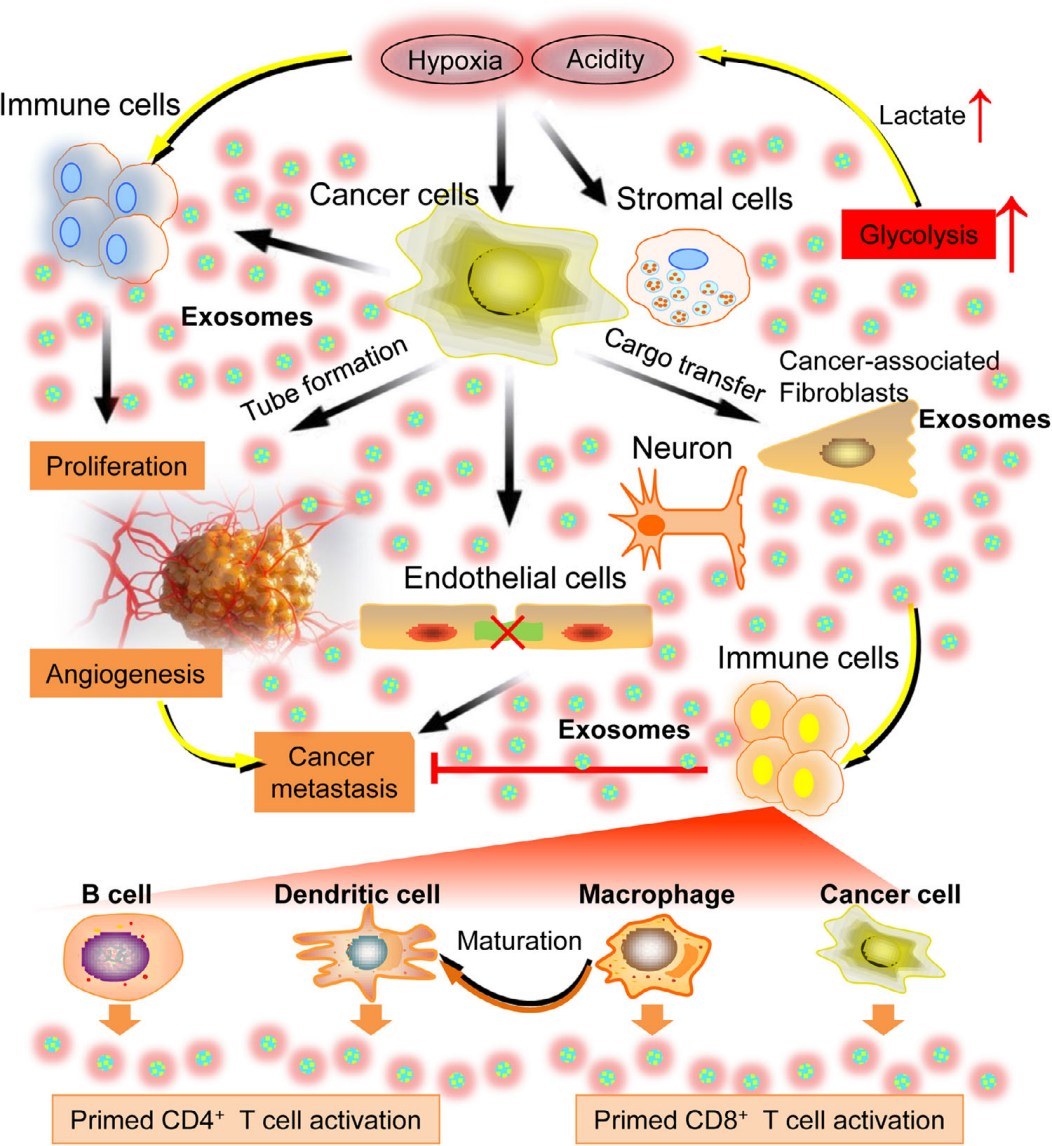
Modulation of various events in the interaction between nervous system and cancers mediated by exosomos. Cancer cell‐derived exosomes communicate with both autologous cancer cells and various other normal cells, and participate in different biological phenomena including immunoregulation, cancer metastasis, angiogenesis and proliferation by altering the metabolic status of recipient cells including enhanced glycolysis and lactate production. Further, acidity or hypoxia could lead to increased exosomes produced by cancer cells and vice versus, cancer cell‐derived exosomes cause changes in the surrounding microenvironment including development of an acidic or hypoxia extracellular environment, leading to cancer metastasis. Cancer cell‐derived exosomes also activate the differentiation of fibroblasts into cancer‐associated fibroblasts. Besides, the potential application of exosomes in immunotherapy has also been validated. Schematic representation has been shown to elucidated the immunostimulatory effects by the release of exosomes from B cells, dendritic cells, macrophages, and cancer cells, which can be employed in strategies for exosome‐based cancer immunotherapy.

Metastasis serves as the primary contributor to mortality in the context of cancer progression. The process encompasses a series of sequential stages, including invasion, intravasation, circulation, extravasation, and proliferation at a further location.^114^, ^115^, ^116^ Interestingly, exosomes have the potential to serve as a linking factor that connects the neurological system and cancer at various stages of the process (see Figure 5). During the process of invasion, the induction of EMT occurs, which leads to a reduction in cell adhesion, degradation of the ECM, and promotion of cell migration. As an example, it has been shown that exosomes found in the nervous system allow breast cancer cells to transfer miR‐9 molecules to NF. This transfer leads to a transformation of the NFs into CAFs, which in turn promotes the remodeling of the ECM via the activation of collagens, metalloproteinases, and fibulins. Furthermore, it has been observed that NFs possess the ability to secrete exosomes carrying miR‐9, which subsequently leads to the suppression of E‐cadherin expression in tumor cells. This, in turn, promotes the invasion and migration of those tumor cells.^117^ During the process of intravasation, exosomes originating from the nervous system disrupt the endothelium, therefore increasing vascular permeability and encouraging tumor cell invasion through blood and lymphatic arteries. Studies conducted both in vitro and in vivo have examined the impact of exosomes originating from metastatic breast cancer cells, which contain TSP1, on human umbilical vein endothelial cells. These studies have demonstrated an enhanced trans‐endothelial migration of tumor cells as a result of the disruption of intercellular junctions. A reduction in the mRNA expression of junction proteins, such as vascular endothelial‐cadherin and zona occluden‐1, has corroborated this disruption.^118^ Moreover, during the process of circulation, tumor cells secrete exosomes that have a great influence on the immune system by suppressing the antitumor functions of natural killer cells and T‐cells.^119^ In general, exosomes derived from the nervous system have the potential to play significant and diverse roles in the malignant advancement of cancer through paracrine, endocrine, and autocrine signaling mechanisms. The subsequent discussion delves into an in‐depth examination and evaluation of significant scholarly works pertaining to the prospective involvement of exosomes derived from the nervous system within the framework of feasible therapeutic approaches.

### 6.2 Targeting nervous system exosomal release and uptake for cancer diagnostics and immunotherapy

The heterogeneity of exosomes poses both methodological hurdles and promising potential for diagnostics and therapeutic procedures.^120^, ^121^, ^122^, ^123^ Numerous studies have provided compelling evidence indicating that malignant brain cancer cells exhibit a notable increase in the release of exosomes into the extracellular environment, which can be attributed, at least in part, to metabolic reprogramming and lactate production. This phenomenon serves to promote tumor survival by facilitating autologous and heterologous interactions between the cancer cells and neighboring cells.^124^, ^125^ Brain cancer‐derived exosomes possess the ability to traverse both the blood–brain barrier (BBB) and the blood–CSF barrier. These exosomes can serve as valuable tools for identifying biomarkers that can be used to monitor the malignant advancement of primary BTs.^126^, ^127^ In recent times, there has been validation through molecular profile investigations that exosomal components, such as nervous system exosomal microRNA and proteins, serve as significant indicators in the detection of tumor malignancy.^128^ The research performed by Thakur et al.^91^ initially demonstrated the efficacy of a label‐free and highly sensitive techniques in detecting and quantifying elevated levels of CD44 and CD133 in exosomes derived from brain cancer cells, which were immune captured from the blood and CSF of a mouse model with brain cancer. The potential utilization of this biosensor as a minimally invasive molecular diagnostic tool for monitoring the progression of brain cancer, akin to a liquid biopsy has been shown.

Moreover, Thakur et al.^127^ were the first to identify that exosomes derived from hypoxic brain cancer exhibited notably elevated levels of monocarboxylate transporter 1 (MCT1) and CD147 (a transmembrane glycoprotein associated with tumors). This finding suggests that these exosomes could serve as accurate biomarkers for monitoring the metabolic reprogramming and malignant progression of primary brain cancer. It is noteworthy that brain cancer cells have been observed to upregulate the expression of MCT1 and CD147, as well as promote their localization at the plasma membrane. This alteration facilitates the extrusion of intracellular lactate, hence supporting the sustenance of uninterrupted glycolysis. The aforementioned phenomenon results in the buildup of lactate inside the TME.^129^ The starving cancer cells in the brain and the stromal cells in the hypoxic TME can uptake extracellular lactate, which leads to the production of adenosine triphosphate. This process ultimately establishes metabolic coupling among various cells in the vicinity.^130^ Recent studies have indicated that lactate present in the TME can fulfill several roles, including serving as an energy source and precursor for biosynthesis. Additionally, lactate has been identified as a signaling molecule that contributes to the promotion of tumor development.^131^ According to the theory known as the “neuron‐astrocyte lactate‐shuttle,” lactate is released by ACs into the extracellular space, where it may be absorbed and processed by neurons. This metabolic mechanism allows neurons to use lactate aerobically, hence supporting essential cellular activities that are crucial for maintaining brain homeostasis.^132^

The inhibition of the release or absorption of exosomes in the nervous system has been identified as a potential strategy to impede the progression of metastasis associated with cancer.^133^ Several in vivo and clinical studies have demonstrated the involvement of the syndecan heparan sulfate proteoglycans (heparanase/syndecan‐1 axis) in exosome production and cancer progression. Consequently, these factors might potentially serve as targets for therapeutic interventions aimed at mitigating cancer development.^134^, ^135^, ^136^ The latest evidence indicates that the presence of an acidic extracellular environment has the potential to induce changes in the generation of exosomes within malignant cells. In an acidic environment, melanoma cells exhibit an increased exosome release compared with normal physiology, indicating the significance of pH in the TME in regulating exosomal trafficking in malignant cells.^137^ Other types of cancers, such as osteosarcoma, as well as breast, colon, and prostate cancer, have also been documented to exhibit comparable characteristics. There is an accepted opinion that the increased secretion of exosomes under low pH settings has the potential to alleviate the intracellular buildup of harmful compounds.^138^ For instance, the utilization of proton pump inhibitors has been observed to effectively decrease the concentration of exosomes in experimental models of cancer.^139^ Furthermore, it has been documented that the proteins RAB27A and RAB27B play a pivotal role in the process of exosome generation and release, as evidenced by the inhibition of exosome release upon their knockdown.^140^, ^141^ A previous study has shown the inhibition of exosome release through the interaction of the RAB27A–JFC1 complex with two drugs, namely Nexinhib4 and Nexinhib20.^142^ Previous research has shown that the treatment with a therapeutic antibody diminished the secretion of exosomes originating from tumors. This reduction in exosome release was found to correspond with a decrease in the metastasis of breast cancer within an in vivo experimental model. These findings indicate the potential use of this approach in the field of cancer treatments.^143^ Moreover, the downregulation of MCT1 and CD147 resulted in a decrease in exosomal release, whereas their upregulation led to a large rise in exosomal secretion. This observation implies that MCT1 and CD147 have the potential to serve as viable targets for anticancer interventions aimed at inhibiting exosome secretion.^127^ Moreover, the absorption of exosomes by recipient cells plays a pivotal role in facilitating subsequent signaling cascades. The internalization process is dependent on various molecules and glycoproteins present on both the exosomal membrane and the receiving cell.^140^ Several exosomal uptake inhibitors have been produced, such as dynasore, heparin, amiloride, and chlorpromazine.^140^ Heparin functions as an inhibitor of endocytosis by obstructing the interaction between heparin sulfate proteoglycans, which are located on the plasma membrane.^144^ Chlorpromazine, a pharmaceutical compound, exerts its inhibitory effects on the formation of clathrin‐coated pits through the modulation of several receptors such as histamine, dopamine, and serotonin. Consequently, it functions as an inhibitor of clathrin‐mediated endocytosis.^145^ Amiloride selectively interacts with the sodium/proton exchanger, hence impeding the process of macropinosome production.^146^, ^147^ Dynasore is a selective inhibitor of dynamin 2, a protein that plays a crucial role in clathrin‐mediated and caveolin‐based endocytosis.^148^ In general, the strategic approach of directing the release of exosomes from donor cells within the nervous system and their subsequent absorption by recipient cells presents a promising avenue to be explored for cancer therapeutics.

Much research has been conducted to create vaccinations for cancer therapies, commonly known as active‐specific immunotherapy or therapeutic cancer vaccines.^149^ In recent investigations, it has been shown that exosomes derived from the nervous system exhibit significant potential in the field of cancer immunotherapy, presenting a viable option for the development of an efficacious cancer vaccine. Figure 5 presents a visual representation of the comparative impact of nervous system exosomes derived from different cell types, encompassing immune cells, cancer cells, and normal cells. These exosomes have the potential to be used in exosome‐based cancer immunotherapy. The potential utility for exosomes produced from B‐cell lymphoma cells (referred to as BL‐EXO) in immunotherapy has also been investigated. B‐cells secrete exosomes that contain major histocompatibility complex (MHC)‐II peptide complexes, therefore enhancing the presentation of antigens to primed CD4^+^ T lymphocytes.^150^ Previous research has demonstrated that clonal proliferation of T‐cells was induced by the presence of BL‐EXO. Additionally, it has also been observed that BL‐EXO may enhance the production of interleukin (IL)‐6 and tumor necrosis factor)‐α, while concurrently suppressing the expression of immunosuppressive cytokines IL‐4 and IL‐10.^151^ BL‐EXO that have been subjected to heat shock have a higher abundance of heat‐shock protein (HSP) 60 and HSP90 proteins. Additionally, these heat‐shocked BL‐EXO demonstrate an increased presence of immunogenic molecules and possess the ability to stimulate CD8^+^ T‐cells, resulting in the induction of antitumor responses.^152^

Dendritic cells (DCs) play a critical role in tumor immunity by effectively capturing and presenting tumor antigens, rendering them indispensable in the development of immunotherapeutic strategies for combating cancer. Nevertheless, the efficacy of DCs in combating tumors is suboptimal due to their limited ability to induce an immunogenic response, inadequate absorption of antigens, and insufficient activation of T‐cells.^153^ In recent reports, there has been mention of the possible consequences of antigen presentation concerning nervous system exosomes derived from DCs.^154^ DCs secrete significant amounts of exosomes, which effectively elicit antitumor responses. This efficacy can be attributed to the high concentration of MHC I and II, HSP, and CD86 found in DC‐derived exosomes. These components play a crucial role in activating CD4^+^ and CD8^+^ T‐cells.^155^, ^156^, ^157^ Furthermore, exosomes produced from DCs can stimulate CD8^+^ and CD4^+^ T‐cells, resulting in the induction of antitumor responses through the presence of exosomal CD80 and IL‐2 in an in vivo experiment.^158^, ^159^ Another research presented evidence indicating that exosomes derived from DCs can induce the production of CD8^+^ T‐cells expressing interferon (IFN)‐γ in mice with hepatocellular carcinoma. This induction is accompanied by increased levels of IFN‐γ and IL‐2, as well as decreased levels of CD25^+^FOXP3^+^ T_regs_, IL‐10, and transforming growth factor‐β.^160^ Although it has been believed that DC‐derived exosomes loaded in MHC trigger a T‐cell response, other studies have shown that in the presence of complete antigens, an MHC‐independent T‐cell response is elicited.^161^ In brief, exosomes generated from DC were able to elicit an immunological response. Exosomes derived from DCs, a subset of antigen‐presenting cells crucial for the functioning of the adaptive immune system, bear either MHC class I or MHC class II peptide complexes. This enables the identification of CD4^+^ or CD8^+^ T‐lymphocytes. In addition, it has been observed that macrophages secrete exosomes containing pathogen‐related antigens when exposed to pathogens like bacteria. This process aids in the maturation of DCs and the subsequent release of proinflammatory cytokines.^162^, ^163^, ^164^ In addition to immune cells, exosomes generated from cancer cells have the potential to induce immunological activation or suppression.^165^, ^166^ It is worth noting that exosomes generated from normal cells exhibit diverse immune‐modulatory functions, hence promoting normal physiological processes.^167^

In general, a range of pharmaceutical substances has been identified to impede the release or absorption of pro‐oncogenic exosomes of the nervous system in the TME. These substances have the potential to be explored as innovative cancer treatments.^168^, ^169^ Another burgeoning field of study pertaining to exosomes inside the nervous system, which has drawn significant interest, is their utilization in immunotherapy to develop prospective cancer vaccines. Numerous kinds of cells, including B‐cells, cancer cells, macrophages, DCs, and normal cells, have been utilized for the isolation of exosomes in the context of cancer immunotherapy. However, it is important to note that each of these sources of exosomes has distinct benefits and drawbacks when it comes to the development of cancer vaccines.

## 7 CANCER THERAPIES’ IINFLUENCE ON THE NERVOUS SYSTEM

Elucidating the mechanisms by which cancer therapy influences nervous system function is central to understanding the bidirectional interactions between neural and cancer cells. Chemotherapy has improved cancer patients’ year‐on‐year survival rates, from 50% in 1 to 10 and over 40 years.^170^ However, it has many detrimental side effects, including fatigue, hair loss, nausea, and cognitive impairment, which reduce survivors' standard of life.^171^ One of the side effects includes multiorgan toxicity. In the brain, chemotherapeutic compounds and chimeric antigen receptor T cell immunotherapy cause cytokine release syndrome,^172^ which is manifested with increased cytokine levels, which disrupts the permeability of the BBB, infiltrating neurotoxic CD8 positive T cell and causes cognitive impairment.^173^ Although different anticancer drugs have different mechanisms of action, however, they may share common neurotoxicity pathways. Chemotherapy mechanisms influencing nervous system primarily include neuroinflammation, impaired neurogenesis, reduced neurotransmission, oxidative stress, and BBB disruption.^174^ The anticancer therapy mechanisms causing nervous system dysregulations are heterogeneous and diverse; however, some overlaps exist that could be targeted as bio‐indices for efficient drug interventions.^174^ The coming 5 years are promising for novel and repurposed drug therapies with beneficial effects. Clinicians should routinely perform objective neural functional tests before and during cancer therapies. Additionally, translational research should elucidate how therapies other than chemotherapy influence cognition, and clinical trials should be performed to assess novel therapeutics.

Elucidating how chemotherapies alter nervous system activities is linked with the knowledge of the bidirectional communication of neural and malignant cells. Since cancer treatments are most focused on enhancing survival and standard of life, cognitive dysfunction is arguably the most important side effect. There is now a clearer epidemiologic picture, with increased self‐reported rates being confirmed by objective neuropsychological analyses, but data for different cancer and their therapies are required.^174^ Traditional cancer therapies, including chemotherapies and radiation, have long‐lasting lethal impacts on nervous system activities, evident as cancer therapy‐associated cognitive disruption (impaired memory, attention, multitasking, and sometimes enhanced anxiety) and as peripheral neuropathies (motor weakness, sensory loss, or pain).^174^ Similar prolonged nervous system impact by novel targeted and cancer immune therapies require attention. Cancer therapies differentially influence cognition and the nerve types impacted in chemotherapy‐induced peripheral neuropathy. The primary molecular and cellular etiologies of neural toxicity mediated by cancer therapies are being researched, and novel neuroprotective or neural regeneration therapies are also emerging.^6^, ^175^ However, further elucidation requires further elucidation of how much chemotherapy‐mediated neuropathy regulates nerve–cancer communication to inhibit malignant growth and the potential benefits of therapy‐mediated neurotoxicity on the antineoplastic influence of radiation and chemotherapy. A comprehensive investigation of pathways and the influence of cancer therapy‐mediated neurotoxicity is required for optimizing therapeutic strategies with minimum neurological side to treat cancer.

## 8 CLINICAL IMPLICATIONS FOR POTENTIAL TREATMENT STRATEGIES TARGETING CANCER NEUROSCIENCE

**FIGURE 6:**
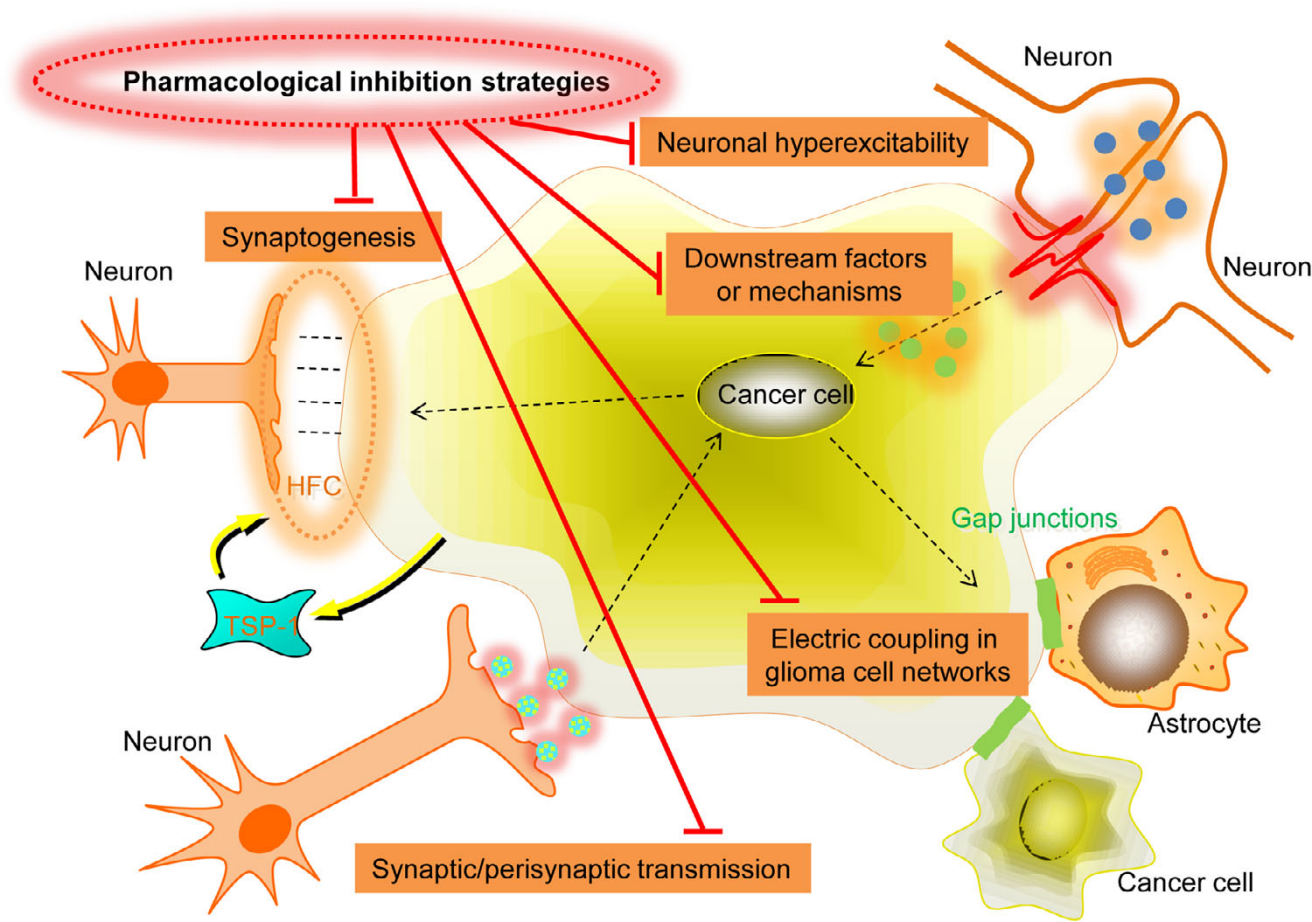
Potential neuron‐to‐brain tumor synaptic communication (NBTSC) targeting therapies. Synaptic and perisynaptic neuronal and brain tumor cell interactions and downstream influence within and across tumor networks are schematically illustrated. Briefly, neuron‐to‐GBM synaptogenesis suppression, synaptic and perisynaptic signal transmission inhibition, GBM cell networks electric coupling disruption, NBTSC downstream mechanisms inhibition, and neuronal hyperexcitability repression after brain tumor stimulation are promising treatment targets against NBTSC. Key NBTSC mechanisms that can be therapeutically targeted are depicted in boxes. TSP‐1, thrombospondin/thrombin sensitive protein 1.

Although the field of cancer neuroscience is still in its infancy, recent studies have clearly confirmed the importance of elucidating nervous system–cancer interactions. The identification of synaptic interactions between BT cells and neurons has fundamentally altered our knowledge of GBMs or brain metastases and indicated how these tumors could invade complex neuronal networks. Therefore, determining new therapeutic avenues against neuron‐to‐BT synaptic communication (NBTSC) require urgent attention. Studies on NBTSC‐targeting therapies can indicate essential targets of yet incurable brain cancers.^174^ Exploiting NBTSCs’ properties and functions will allow multiple avenues for identifying new antitumor treatments (Figure 6). Currently, the treatment is either symptomatic or aimed at alleviating tumor mass and not the communication of cancer cells with adjacent tissues. Theoretically targeting the factors that promote progression and direct neuronal synaptic interference with cancer cells could help develop neuroactive drugs.^176^ This is an updated review indicating potential approaches targeting NBTSC.

First, it is worth noting that inhibiting neuron‐to‐GBM synaptogenesis has great potential. It can be done by preventing new malignant synapses. CNS synaptogenesis is markedly complex and non well understood.^177^ It comprises pre‐ and postsynaptic protein expression, approximating pre‐ and postsynaptic membrane, and the subsequent maturation of the synapse. Neuronal synaptic contacts can be stimulated by filopodia from the growth cones of axons or dendrites that randomly sprout out.^178^ After approximation, adhesion molecules stabilize the cell membranes. The most crucial members, neurexins and neuroligins at the pre‐ and postsynaptic sites, respectively, induce the differentiation of opposing sides.^179^, ^180^ Venkatesh et al.^7^ revealed that soluble neurexins or ADAM10 inhibitors (sheds neuroligin‐3) are good approaches to reducing malignant synaptogenesis. Furthermore, glial cells induce neuronal synaptogenesis by releasing stimulating factors like TSP.^181^ Moreover, gabapentin antagonizes the binding of TSP with its receptor, the calcium channel auxiliary protein α2δ, thus suppressing excitatory synaptogenesis.^182^ Pregabalin also binds α2δ, and so also inhibits synaptogenesis.^183^ Whether pregabalin and gabapentin also influence experimental GBM models and have antitumor effects by inhibiting NBTSC is unknown and requires further preclinical studies (Figure 6).

Second, synaptic and perisynaptic signal transmission inhibition is also an efficient treatment strategy. Researchers have worked hard since the 1980s to develop noncompetitive and competitive AMPAR suppressors.^184^ Since early competitive AMPAR suppressors demonstrated unfavorable pharmacological effects,^185^ noncompetitive AMPAR suppressors are now being elucidated. Talampanel and Perampanel are noncompetitive AMPAR suppressors that have indicated potential results in early epilepsy and GBM trials.^186^, ^187^, ^188^, ^189^, ^190^ Currently, a randomized placebo‐controlled trial on AMPAR antagonists is being carried out. The antagonist has an acceptable pharmacokinetic profile and targets NBTSC at the tumor infiltration site, with standard care (resection, radio‐ and chemotherapy) that effectively treats primary tumor mass, which is significant for elucidating the potential of AMPAR suppression in GBMs. Furthermore, neurotransmitter receptors other than AMPAR, such as NMDARs, are also essential in NBTSC. However, targeting NMDAR have many challenges due to a small therapeutic window in humans and can result in lethal effects.^191^ But recently, allosteric NMDAR modulators^192^ have revealed novel drugs for controlled trials. Therefore, AMPAR or NMDAR suppression can be an efficient GBM therapy that requires validation by a controlled clinical trial.

Third, electric coupling disruption in GBM cell networks could be therapeutic. As stated above, GBM cells communicate with functional networks via gap junctions on tumor microtubes, rendering GBMs more resistant to standard therapies.^28^ Since gap junctions interact with GBM cells electrically, gap junction inhibitors reduced the frequency or SICs amplitude of a single cell and repressed proliferation in the murine model.^7^, ^9^ These inhibitors are also anticonvulsants, potentially alleviating neuronal hyperexcitability and NBTSCs’ downstream network effects.^193^ Tonabersat is a gap junction modulator that treats migraine and is well tolerated.^194^ In 2019, it was assessed as an adjuvant with radiochemotherapy in a rat glioblastoma model and showed reduced tumor growth than radiochemotherapy alone.^195^ However, the effects of gap junction inhibitors such as tonabersat and meclofenamate in humans require future research (Figure 6).

Fourth, blocking downstream NBTSC mechanisms is another potentially effective option. Synaptic input can be translated into calcium transients, activating downstream pathways. Different translation routes exist; for instance, depolarization directly activates voltage‐gated calcium channels (VGCCs) to permit calcium influx or reduced calcium level passing via AMPARCa amplified by calcium‐induced calcium release (CICR) or inositol 1,4,5‐triphosphate‐induced calcium release (IICR). Much research has been done on the inhibitory influence of VGCC blockage on GBM cell growth.^196^ Several VGCC inhibitors exist, including pregabalin and gabapentin, which have an antiepileptic impact (refer to synaptogenesis and hyperexcitability section).^197^, ^198^ CICR and IICR are critically involved in neurons,^199^ glial cells,^200^ and suppressors, including ryanodine and thapsigargin.^201^, ^202^ Further investigations assessing the exact NBTSC downstream mechanisms are crucial.

Last, suppressing neuronal hyperexcitability, thereby inhibiting BT stimulation, is also a potential treatment strategy. Neuronal hyperexcitability might be the primary cause of GBM and brain metastases progression. Therefore, antiepileptic drugs might have profound clinical importance. However, specific antiepileptics for potentially stratified GBM subtypes need systematic analysis in future trials.^173^

Since these interaction pathways are not unique to tumor cells, targeting NBTSC mechanisms while preserving the normal CNS's functional integrity is crucial. Pharmacological AMPARs inhibition has revealed promising data, and further work will identify downstream NBTSC targets and their potential translational relevance. Much research has been done on neurotransmitters like glutamate, and so on, for their activity in BT. Furthermore, the possibility that some influences might be conveyed via NBTSC needs elucidation. Whether synergistic impact exists between NBTSC suppressors and established cytotoxic therapies, including radio‐ and chemotherapy, and whether anti‐NBTSC therapies are better as monotherapies also remain unknown.

## 9 CONCLUSION AND PROSPECT

Overall, both the CNS and the PNS regulate physiological functions and pathophysiological processes of cancers. Based on converging evidence, CNS activity and PNS activity regulate development, plasticity, homeostasis, regeneration, as well as immune function in diverse tissues. As cancer growth and progression subvert mechanisms of development and regeneration, the nervous system may be implicated in all aspects of cancer pathophysiology. Reciprocally, cancer and cancer therapies can also impact or remodel the nervous system, contributing to pathological feedback loops, yielding neurological dysfunction and together driving malignancy. These new viewpoints have culminated in the emergence of cancer neuroscience, leading to more neuroscientists’ efforts directed toward defining and therapeutically targeting nervous system–cancer interactions, both in the local TME and systemically.

There could be various open questions or challenges currently. For example, more efforts should be directed toward clarifying whether nervous system–cancer interactions promote cancer progression/metastasis. And it is unknown whether combining therapies to block these neural–cancer interactions cold be powerfully synergistic for combating therapeutic resistance. Besides, it is still unclear whether targeting nervous system–cancer interactions by itself could be sufficient to eradicate a tumor, or whether this could be a necessary component of effective therapeutic strategies for currently intractable cancers, like high‐grade gliomas and pancreatic cancers. Promisingly, cancer neuroscience holds the promise to elucidate fundamentally new insights into the pathobiology of cancers.

It will not be wrong to say that we are still at the beginning of elucidating how nervous system is associated with cancer initiation, spread, growth, recurrence, and therapeutic resistance. The knowledge of modern neuroscience, electrophysiology, and optogenetics should be used to understand cancer pathophysiology. Furthermore, the type‐specific variability in tissue and tumor requires careful research for each cancer type progression to understand how malignancy and cancer‐mediated nervous system remodeling coevolve. Additionally, single‐cell analyses with novel lineage analysis and pluripotent stem cell modeling tools should be utilized to define and link the myriad nerve types with specific cancer phenotypes.^203^

For comprehensive understanding, an interdisciplinary investigation and collaboration between neuroscience, immunology, developmental biology, and cancer biology should be performed. Direct neuron–cancer cell communications and the nervous system's cellular influence in the local stroma, immune, and systemic tumor environment should be focused on. Exciting opportunities exist for cancer biologists in the field of cancer genomics, immuno‐oncology, and precision therapeutics with a new dimension in the armamentarium. Neuroscientists can use sophisticated modern approaches to benefit current therapies and cancer patients. The neural tumor growth regulation still needs attention; early‐phase clinical trials targeting neural pathways that regulate different tumor growth are underway. Focusing specifically on neural–cancer crosstalk will furnish novel strategies and improved outcomes for various difficult‐to‐treat malignancies.

## AUTHOR CONTRIBUTION

Y. L. L., S. Z., and Y. Z. conceived and designed this study. Y. L. L. and S. Z. performed the data analysis and figure plotting. Y. L. L., S. Z., W. W., and Q. C. were involved in writing original draft. Y. L. L. and Y. Z. were responsible for the critical reading of the manuscript. All authors contributed to the article and approved the submitted version.

## CONFLICT OF INTEREST STATEMENT

No potential conflict of interest were reported by the authors.

## ETHICS STATEMENT

Not applicable.

## Data Availability

Not applicable.
